# Meta-analysis Comparing Celecoxib with Diclofenac Sodium in Patients with Knee Osteoarthritis

**DOI:** 10.1093/pm/pnaa230

**Published:** 2020-08-14

**Authors:** Hetao Huang, Minghui Luo, Haodong Liang, Jianke Pan, Weiyi Yang, Lingfeng Zeng, Guihong Liang, Senrong Hou, Jinlong Zhao, Jun Liu

**Affiliations:** 1 Second School of Clinical Medicine, Guangzhou University of Chinese Medicine, Guangzhou, China; 2 Department of Orthopaedics, Second Affiliated Hospital of Guangzhou University of Chinese Medicine (Guangdong Provincial Hospital of Chinese Medicine), Guangzhou, China; 3 Guangzhou Hospital of Traditional Chinese Medicine, Guangzhou, China

**Keywords:** Celecoxib, Diclofenac Sodium, Knee Osteoarthritis, Meta-analysis

## Abstract

**Objective:**

To compare the efficacy and safety of celecoxib and diclofenac sodium in patients with knee osteoarthritis (KOA).

**Methods:**

Clinical controlled trials (CCTs) and randomized controlled trials (RCTs) from online databases comparing the efficacy of celecoxib and diclofenac sodium in the treatment of KOA were retrieved. The main outcomes included the treatment effect, C-reactive protein (CRP) level, erythrocyte sedimentation rate (ESR), visual analog scale (VAS) score, and complication rate. The Cochrane risk of bias (ROB) tool in Review Manager 5.3.5 was used to assess methodological quality.

**Results:**

Twelve studies (N = 2,350) were included in this meta-analysis. The meta-analysis indicated that celecoxib reduced pain more effectively than diclofenac sodium in patients with KOA, as evaluated by the VAS score. In addition, celecoxib has certain advantages in terms of better treatment effects and greater reductions in the ESR, CRP level, and complication rate.

**Conclusions:**

Celecoxib is superior to diclofenac sodium in the treatment of KOA. However, well-designed and high-quality RCTs are still needed.

## Introduction

Knee osteoarthritis (KOA) is the most common degenerative disease that leads to joint pain, loss of function, and disability in the elderly [1]. The main clinical manifestations of KOA are knee pain and dysfunction, which affect the quality of life of the elderly and increase the social and economic burden [2, 3]. With the aging of the global population and the increase in the number of joint injuries, KOA is becoming increasingly more prevalent. According to worldwide estimates, 250 million people are currently affected [1]. An epidemiological survey showed that the global prevalence of KOA is ∼12–35% [4]. KOA has significantly increased health care expenditures and has attracted the attention of governments and public welfare organizations in some Asian countries [5]. The treatment of KOA mainly includes conservative treatment and surgical treatment. Conservative treatment mainly includes oral or topical anti-inflammatory drugs (NSAIDs), intra-articular injection, and physical therapy. Surgical treatment mainly includes knee arthroscopy, osteotomy, unicompartmental knee arthroplasty (UKA), and total knee replacement (TKA) [6].

KOA is a whole-joint disease, and its pathological manifestations are mainly articular cartilage damage, osteophyte formation, degeneration, and damage to the subchondral bone and meniscus [7, 8]. The pathogenesis of KOA is complex and involves multiple factors, such as inflammation and metabolic factors, which ultimately lead to structural damage and failure of the joint. The treatment goals in the management of KOA have been to relieve pain, restore joint function, delay disease progression, and ultimately improve the patient’s quality of life [9]. NSAIDs currently have the most abundant clinical evidence and are the most common prescription analgesics for KOA [10]. They mainly block the metabolism of arachidonic acid (AA) by inhibiting cycloxygenase (COX), reducing the production of prostaglandin (PG), to achieve anti-inflammatory and analgesic effects [11]. NSAIDs are mainly divided into four types: COX-2 selective inhibitors, COX-1 high selective inhibitors, COX-1 low selective inhibitors, and COX nonselective drugs [12]. Most NSAIDs can stimulate the gastrointestinal tract and induce ulcers, and they also affect renal function and platelets [13]. Because selective COX-2 inhibitors do not affect the activity of COX-1, they can significantly reduce the gastrointestinal side effects caused by the inhibition of COX-1. Some studies have demonstrated that specific COX-2 inhibitors can reverse the imbalance of chondroproteoglycan metabolism mediated by inflammatory cytokines, restore the content of chondroproteoglycans, and promote their repair [14]. Thus, they may be able to reduce the symptoms of KOA and reverse the pathological changes at the same time. NSAIDs are effective in pain relief and joint function improvement of patients with KOA. Many guidelines still recommend NSAIDs as the most commonly used drug for patients with KOA.

Celecoxib is a COX-2 selective inhibitor that is commonly used in the clinical treatment of KOA, but diclofenac sodium is not. Based on the mechanism of drug action, celecoxib should theoretically have better efficacy and fewer gastrointestinal adverse reactions. The efficacy and safety of celecoxib and diclofenac sodium in the treatment of KOA have been confirmed by many clinical studies. However, due to the small sample size of a single study and the various biases that may exist during the implementation of the study, it is unclear which is more advantageous in the treatment of KOA. In this study, we searched an authoritative database for high-quality clinical studies of celecoxib and diclofenac sodium in the treatment of KOA and carried out a systematic review and meta-analysis in strict accordance with the Cochrane system model. This study objectively and accurately evaluates the efficacy of celecoxib and diclofenac sodium in the treatment of KOA and compares their efficacy. Finally, this meta-analysis provides reliable evidence-based medical guidance for clinicians and provides a reference for treatment decision-making.

## Methods

According to the Preferred Reporting Items for Systematic Reviews and Meta-Analyses (PRISMA) criteria [15], we created a prospective protocol before the implementation of the study, which scientifically limits the literature retrieval strategy, inclusion and exclusion criteria, literature quality evaluation, outcome index measurement, and statistical analysis methods. The study was approved by the Ethics Committee of the Guangdong Provincial Hospital of Chinese Medicine.

### Search Strategy

Seven databases, namely PubMed, the Cochrane Central Register of Controlled Trials, EMBASE, the China Biology Medicine disc (CBM), the Chinese Scientific Journal Database (VIP), China National Knowledge Infrastructure (CNKI), and the Wanfang Database, were searched from the date of their inception through September 2019. In this study, literature retrieval was conducted using the following search terms individually or in combination: “celecoxib,” “diclofenac sodium,” “knee,” “osteoarthritis,” and “arthritis.” No language exclusions were applied. Manual searches were performed for the references in the identified studies.

### Selection Criteria

Two researchers (HTH, MHL) independently read and screened the titles and abstracts of all retrieved articles and determined whether the paper contained potential data related to this study. In case of disagreement, another author (JL) made the final decision after review.

The inclusion criteria were as follows: 1) RCTs/CCTs comparing the efficacy and safety of celecoxib and diclofenac sodium in the treatment of KOA and 2) the outcome measures included at least one key outcome indicator of the study, such as the treatment effect, ESR, VAS score, CRP level, and complication rate. The complication rate refers to the proportion of complications or adverse reactions of patients after taking drugs, such as the proportion of gastrointestinal adverse reactions, cardiovascular adverse reactions, and liver and kidney dysfunction.

The exclusion criteria were as follows: 1) noncontrolled studies, cohort studies, and retrospective studies; 2) animal experiments, letters, case reports, and editorials; 3) studies from which the data have obvious defects or required data could not be extracted; and 4) studies that used other drugs (e.g., other analgesic medications) that may affect the efficacy judgment during the treatment.

### Data Extraction

Two authors (HTH and HDL) extracted the relevant data from the included studies independently according to predefined criteria. The main information extracted includes the date of publication, authors’ names, sample size, sex ratio of patients, average age of patients, follow-up time, and clinical results. In the case of incomplete data in the paper, we contacted the corresponding author or other appropriate author for details. The reasons for exclusion were recorded.

### Quality Assessment

Two researchers (HTH, JKP) independently evaluated the methodological quality of each included study in strict accordance with the standards recommended by the Cochrane Handbook [16]. In case of disagreement, a discussion occurred with the third reviewer (JL) until a consensus was reached. The risk of bias in each study was determined by assessing the implementation of randomization, the concealment of allocation schemes, the use of blinding methods, the integrity of data, outcome reporting, and other biases.

### Statistical Analysis

In this study, Stata 14.0 was used for data statistical analysis, and Review Manager 5.3.5 software (Cochrane Collaboration, Oxford, UK) was used to assess the bias risk of the included studies. According to the characteristics of the data extracted in the study, continuous variable data were expressed as the standard mean differences (MDs) with 95% confidence intervals. Differences in categorical variables are expressed as odds ratios (ORs) and 95% CIs. Initially, the fixed-effect model was used to evaluate the results of the study, unless the heterogeneity tests indicated that the *I*^2^ statistic was ≥50% and substantial heterogeneity existed between studies; in this case, the reasons for this heterogeneity were determined, and a random-effects model was used for comparison.

### Ethics Approval

The systematic review and meta-analysis are secondary studies of published clinical studies in the database. This paper does not contain any studies conducted by the author on human participants or animals, so ethical approval was not required.

## Results

### Description of the Included Studies

**Figure 1.**
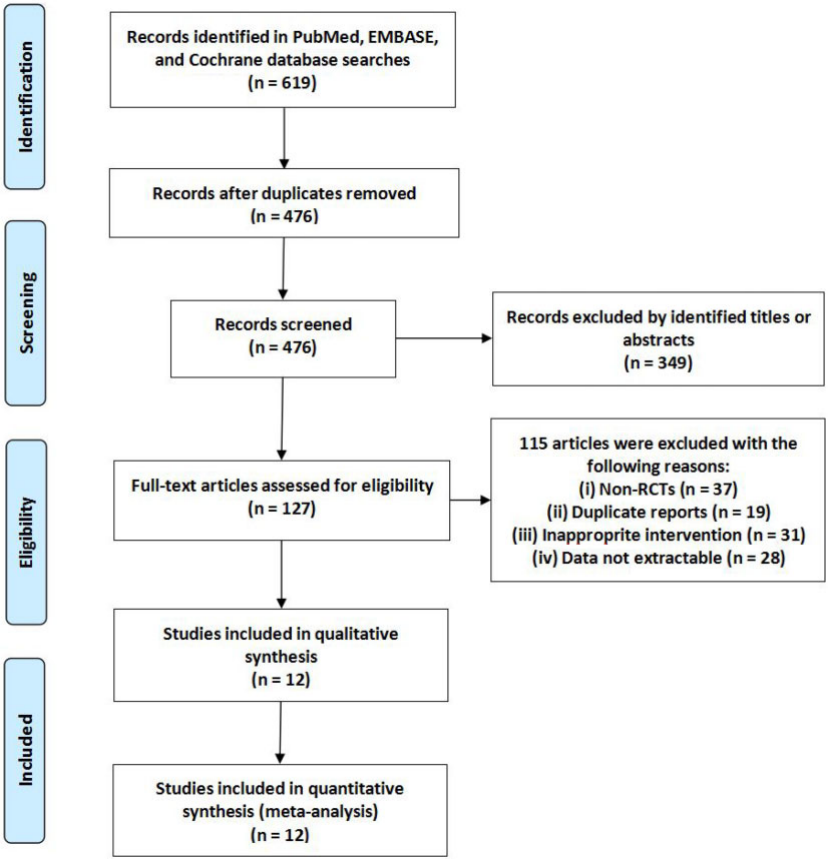
Flow diagram of study selection.

The results of the search strategies are shown in Figure 1. A total of 619 related studies were obtained from the preliminary inspection. After the title and abstract were screened, 127 potential studies with great relevance were obtained. According to the inclusion and exclusion criteria, 115 studies were excluded. Finally, this meta-analysis included 12 studies [17–28].

### Study Characteristics

The characteristics of the 12 studies [17–28] are described in Table 1. A total of 2,350 individuals were included in these studies, and the sample sizes of all included studies were >50. A total of 1,189 individuals were included in the celecoxib group, and 1,161 individuals were in the diclofenac sodium group. Regarding the evaluation index, eight studies [17, 19–24, 28] chose treatment effect, 12 studies [17–28] chose complication rate, five studies [18–20, 26, 28] chose VAS score, one study [19] chose the Lequesne score, one study [19] chose the Western Ontario and McMaster Universities Osteoarthritis Index (WOMAC) score, four studies [20, 22, 23, 28] chose the ESR, and two studies chose CRP level (Table 1) [22, 23].

**Table 1.** Characteristics of the included studies

Authors	Year	Country	Study Type	Sex (M/F)		Age, y		Participants		Outcomes	Follow-up
				Celecoxib	Diclofenac	Celecoxib	Diclofenac	Celecoxib	Diclofenac		
Xiao [17]	2016	China	CCT	21/24	23/22	66	64	45	45	①②	3 mo
Zhou [18]	2002	China	CCT	9/47	5/25	60.4	58.9	56	30	②③	6 wk
Yang [19]	2014	China	CCT	62/50		58.4 ± 4.7		56	56	①②③④⑤	12 wk
Ai [20]	2017	China	RCT	44/36	46/34	56.18 ± 2.25	56.12 ± 2.43	80	80	①②③⑥	6 wk
Xu [21]	2003	China	CCT	22/38		58.2		20	20	①②	6 wk
Li [22]	2016	China	CCT	30/19	27/22	71. 45 ± 8. 84	70. 06 ± 9. 78	49	49	①②⑥⑦	3 mo
Tan [23]	2014	China	CCT	13/36	12/37	60.86 ± 3.52	60.36 ± 3.25	49	49	①②⑥⑦	3 mo
Wu [24]	2007	China	CCT	23/37	22/38	59.5	58.5	60	60	①②	5 wk
Le Dahlberg [25]	2009	Sweden	RCT	149/314	141/321	71 ± 7.0	71 ± 7.3	458	458	②	4 wk
Mani [26]	2012	Kerala	CCT	25/0	25/0	49.88 ± 5.74	50.08 ± 5.56	25	25	②③	7 d
McKenna [27]	2001	UK	CCT	64/137	76/123	61.9	62.7	201	199	②	6 wk
Yu [28]	2018	China	CCT	45/45	48/42	60.4 ± 3.2	59.5 ± 3.6	90	90	①②③⑥	6 wk

① Treatment effect; ② complication rate; ③ VAS score; ④ Lequesne score; ⑤ WOMAC score; ⑥ ESR (mm/h); ⑦ CRP (mg/L).

CCT = clinical controlled trial; CRP = C-reactive protein; ESR = erythrocyte sedimentation rate; RCT = randomized controlled trial; VAS = visual analog scale; WOMAC = Western Ontario and McMaster Universities Osteoarthritis Index.

### Assessment of the ROB

**Figure 2.**
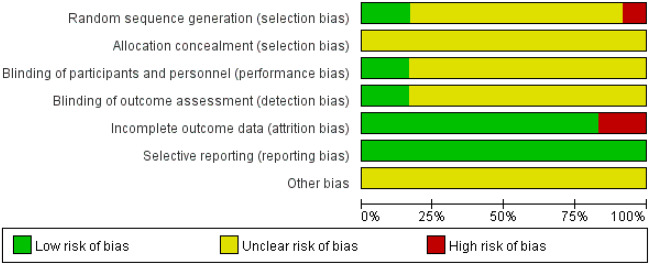
Risk of bias graph.

**Figure 3.**
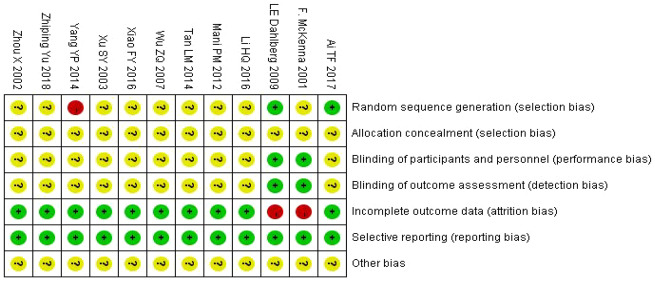
Risk of bias summary.

Figure 2 shows a summary of the ROB assessment for all included studies. 1) With regard to the random sequence generation methods, 12 studies [17–28] mentioned random grouping, nine of which [17, 18, 21–24, 26–28] did not mention specific allocation schemes and were rated as having an unknown risk; one study [19] mentioned grouping according to admission order and was rated as having a high risk; and two studies [20, 25] mentioned grouping according to a random number table and were rated as having a low risk. 2) With regard to allocation concealment, 12 studies [17–28] did not mention the method of allocation concealment, and all were rated as having an unknown risk. 3) Regarding the blinding method used for the study subjects and the implementation of the treatment programs, two studies [25, 27] mentioned the method of blinding and were rated as having a low risk, while the other 10 studies [17–24, 26, 28] did not mention the blinding method and were rated as having an unknown risk. 4) The blinding method used for the measurement of the results was mentioned in two studies [25, 27], which were rated as having a low risk; the other 10 studies [17–24, 26, 28] did not mention the blinding method, so they were rated as having an unknown risk. With regard to incomplete data, two studies [25, 27] lacked data and were rated as having a high risk; the other 10 studies [17–24, 26, 28] all had complete data, so they were rated as having a low risk. None of the studies included had selective reporting of the results [17–28], so they were rated as having a low risk. All studies included were rated as unknown for other risks of bias (Figures 2–3).

### Outcomes of the Meta-analysis

#### Treatment Effect

Treatment effect is usually expressed by the total efficacy rate after drug treatment. The evaluation of the total efficacy rate was based on the Guiding Principles for Clinical Research of New Chinese Medicine formulated by the State Food and Drug Administration of China [29]. The efficacy rate was graded into four categories: clinically controlled, significantly improved, improved, and ineffective. The efficacy rate was calculated as follows: (pretreatment score − post-treatment score)/pretreatment score × 100%. The total efficacy rate was calculated as follows: (number of clinically controlled + number of significantly improved + number of improved)/total number of cases × 100% [29].

**Figure 4.**
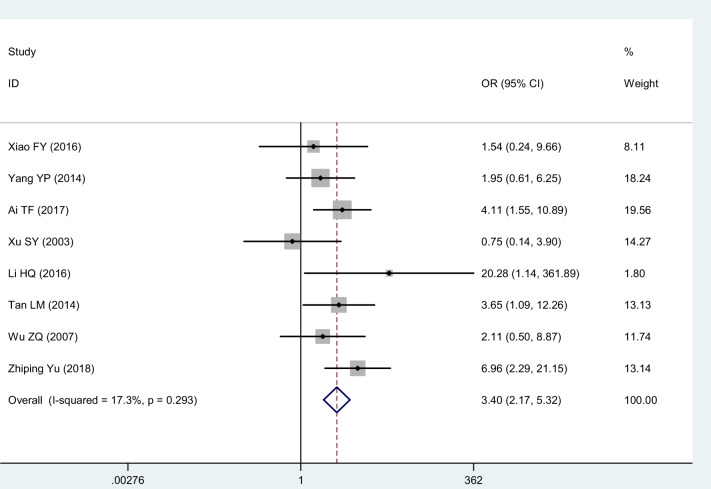
Forest plot of the effect of celecoxib vs the effect of diclofenac sodium on the treatment effect.

Eight studies [17, 19–24, 28] selected the treatment effect as the evaluation index. The meta-analysis showed that (χ^2^ = 8.46, *I*^2^ = 17.3%) had good statistical homogeneity, so a fixed-effects model was adopted. The combined effect (OR = 3.40, 95% CI = 2.17 to 5.32, *P* ≤ 0.001) was statistically significant. It can be concluded that celecoxib is better than diclofenac sodium for the treatment of KOA (Figure 4).

#### VAS Scores

**Figure 5.**
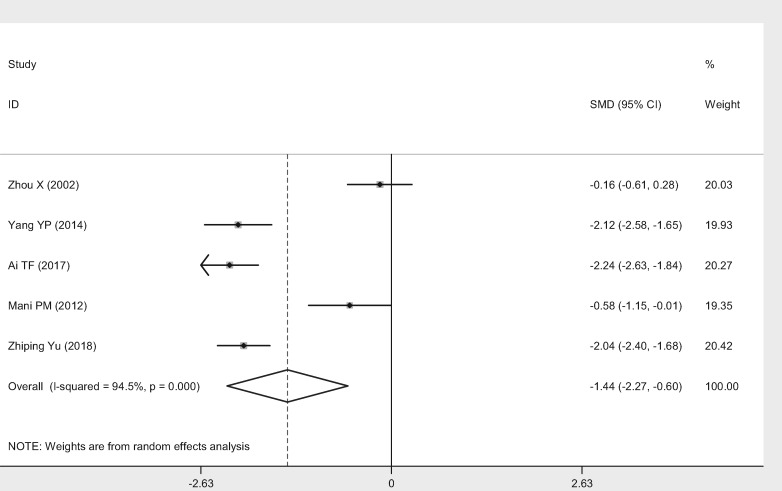
Forest plot of the effect of celecoxib vs the effect of diclofenac sodium on visual analog scale scores.

Five studies [18–20, 26, 28] selected the VAS score as the evaluation index. The meta-analysis results showed that the χ^2^ of 73.27 (*I*^2^ = 94.5%) was highly heterogeneous, so a random-effects model was adopted. The combined effect (SMD = −1.44, 95% CI = −2.27 to −0.60, *P* ≤ 0.001) was statistically significant. Celecoxib was considered more advantageous than diclofenac for reducing the VAS score (Figure 5).

#### ESR

**Figure 6.**
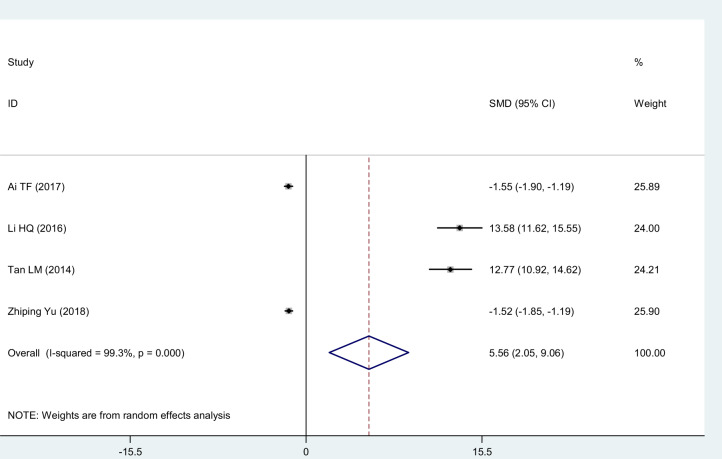
Forest plot of the effect of celecoxib vs the effect of diclofenac sodium on the erythrocyte sedimentation rate.

Four studies [20, 22, 23, 28] selected ESR as the evaluation index. The meta-analysis showed that the χ^2^ of 443.5 (*I*^2^ = 99.3%) had substantial heterogeneity, so a random-effects model was adopted. The combined effect (SMD = 5.56, 95% CI = 2.05 to 9.06, *P* = 0.002) was statistically significant. Celecoxib was considered more advantageous than diclofenac for reducing the ESR (Figure 6).

#### CRP

**Figure 7.**
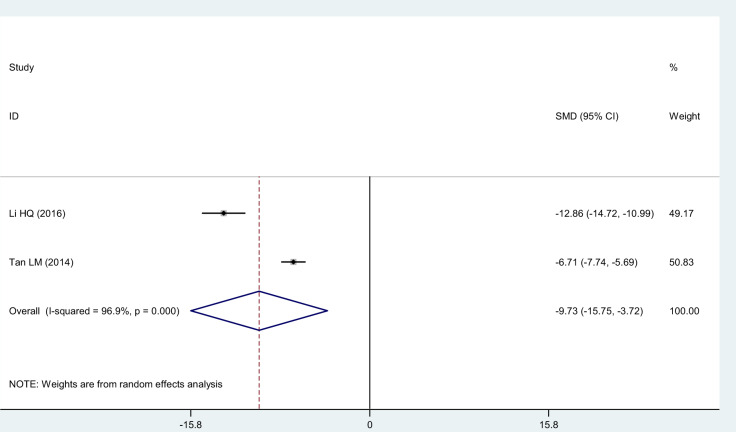
Forest plot of the effect of celecoxib vs the effect of diclofenac sodium on the C-reactive protein.

Two studies [22, 23] chose CRP level as the evaluation index. The meta-analysis results show that the χ^2^ of 32.04 (*I*^2^ = 96.9%) was highly heterogeneous, so a random-effects model was adopted. The combined effect (SMD = −9.73, 95% CI = −15.75 to −3.72, *P* = 0.002) was statistically significant. Celecoxib was considered more advantageous than diclofenac in reducing the CRP level (Figure 7).

#### Complication Rate

**Figure 8.**
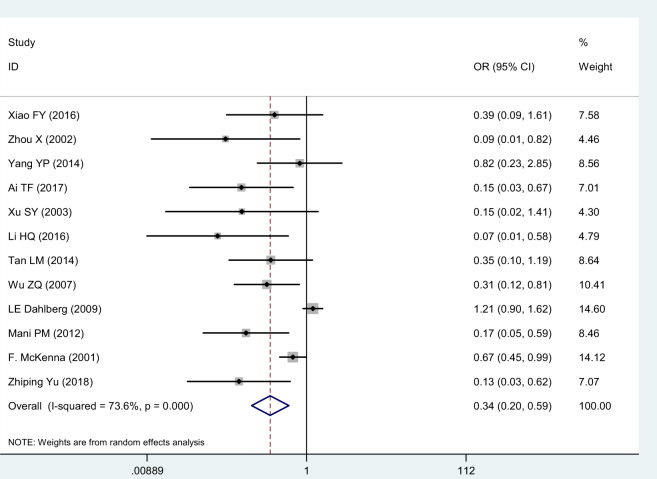
Forest plot of complication rate.

**Figure 9.**
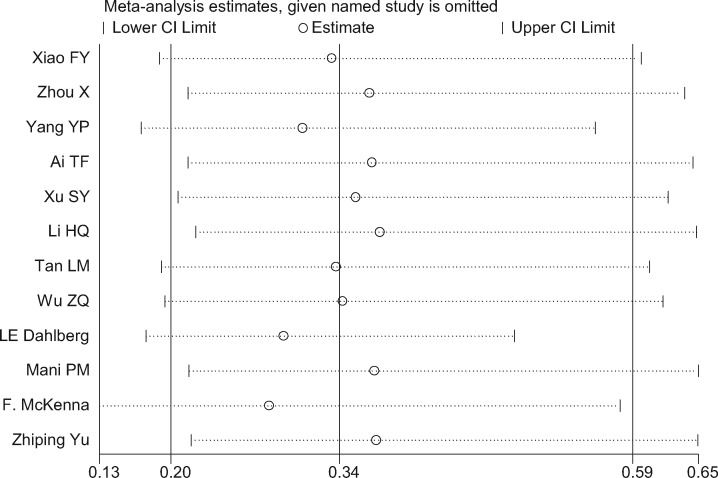
Forest plot of sensitivity analysis.

**Figure 10.**
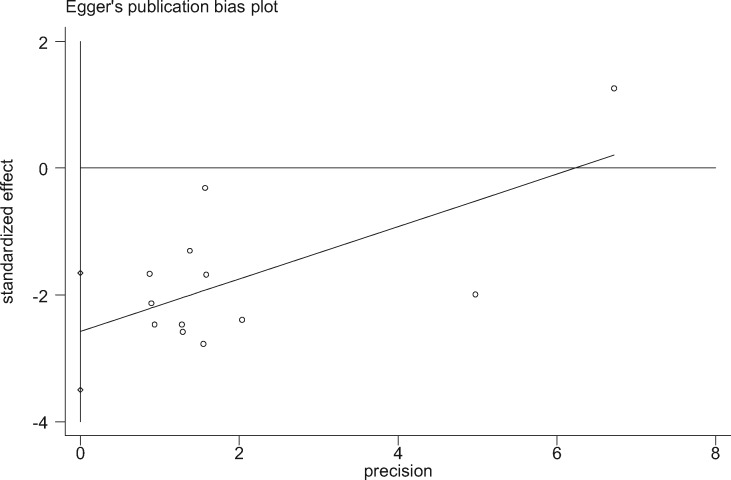
Egger test regression diagram.

Twelve studies [17–28] chose the complication rate as the evaluation index. The meta-analysis results showed that the χ^2^ of 41.68 (*I*^2^ = 73.6%) was significantly heterogeneous, so the random-effects model was adopted. The combined effect (OR = 0.34, 95% CI = 0.20 to 0.59, *P* ≤ 0.001) was statistically significant, suggesting that celecoxib was more effective at reducing complications than diclofenac sodium (Figure 8). The degree of interstudy heterogeneity was large, so a sensitivity analysis was performed that showed that all study point values fell within the 95% CI of the final result. Regardless of which study was excluded, the result was minimally impacted (Figure 9). In the analysis of publication bias, ≥10 studies were included, and Egger’s test was used (*P* ≤ 0.001). The 95% CI (−3.50 to −1.65) did not contain 0, indicating that publication bias was likely, probably due to the low quality of the included studies and the differences in sample sizes (Figure 10).

## Discussion

KOA is one of the most common chronic progressive diseases in the world and is mainly characterized by the degeneration, destruction, and hyperosteogeny of articular cartilage [30, 31]. The pathogenesis of KOA is complex. Although many researchers have been exploring the pathological mechanism of KOA for a long time, its specific pathogenesis remains unclear [32–37]. A total of 250 million people worldwide are affected by OA. The incidence rate of KOA was 17%. OA accounted for 3.9% of the world’s disabled population in 2015. It is expected that OA will become the fourth major cause of disability by 2020. With the aging of the population, KOA will significantly increase medical expenditures, resulting in a significant economic burden to global society [38–40]. Medical expenses related to OA in high-income countries account for 1% to 2.5% of the GDP.

Treatment of KOA mainly includes surgical and nonsurgical treatment. The clinical effect of surgical treatment, including knee arthroscopy, osteotomy, and knee arthroplasty, is more accurate. Nonsurgical treatment can delay the progression of disease and improve the function of the knee joint by health education, and it includes the use of medications to control pain (such as NSAIDs) and physical therapy [41]. NSAIDs are one of the most commonly used nonsurgical therapies for the treatment of KOA, which has good anti-inflammatory and analgesic effects. This type of drug can control arthritis by blocking the pathway of cyclooxygenase and lipoxygenase reversibly, as well as by blocking proinflammatory agents such as prostaglandins and leukotrienes.

Selective COX-2 inhibitors have satisfactory anti-inflammatory and analgesic effects, but they can also cause many adverse reactions, such as gastrointestinal events and cardiovascular disease [42–45]. Rofecoxib was withdrawn from the market in 2004 due to its significant gastrointestinal reactions and cardiovascular toxicity [46, 47]. Cyclooxygenase is the rate-limiting enzyme that plays an important role in the metabolism of arachidonic acid. There are three subtypes of COX: COX-1, COX-2, and COX-3. COX-1 is constitutively expressed and widely exists in most tissues and all types of cells. In general, the concentration of COX-1 remains stable. Under the stimulation of hormones, cytokines, or growth factors, its activity can be increased by two- to fourfold [48]. The function of COX-1 is to protect gastrointestinal mucosa, regulate renal blood flow, balance water and electrolytes, prevent platelet aggregation and maintain normal hemostasis, which plays an important role in maintaining homeostasis. COX-2 is an important inducible enzyme in the process of inflammation. Also known as inflammatory COX, it is mainly located in the nuclear membrane, and it is not expressed in most tissues in the physiological state. The high expression of PGE2, PGI2, and PGE1 in inflammatory tissues was induced by stimulation of lipopolysaccharides (LPS) and inflammatory cytokines, which resulted in an increase in the content of PGE2, PGI2, and PGE1 in inflammatory tissues and inflammatory manifestations, such as redness, swelling, and pain. Selective COX-2 inhibitors do not affect the activity of COX-1, so they can reduce the gastrointestinal events caused by the inhibition of COX-1. The expression of COX-2 in the whole upper digestive tract is decreased, and their gastrointestinal toxicity curve is often more tolerable. It is important that the general risk of bleeding, renal injury, and hypertension is relatively the same among different types of NSAIDs. Many previous independent studies have shown that celecoxib is more effective than diclofenac sodium in relieving knee pain and improving joint function in patients with KOA. However, as the previous conclusions are based on independent studies, the level of evidence is relatively low, and high-quality studies to confirm these results are urgently needed.

In this meta-analysis, data from 12 studies showed that pain relief and improvements in blood parameters in the celecoxib group were superior to those in the diclofenac sodium group. In terms of the treatment effect, the celecoxib group was better than the diclofenac sodium group (OR = 3.40, 95% CI = 2.17 to 5.32, *P* ≤ 0.001). With regard to pain relief (VAS scores), the celecoxib group exhibited better relief than the diclofenac sodium group (SMD = −1.44, 95% CI = −2.27 to −0.60, *P* ≤ 0.001). Celecoxib seems to be more effective than diclofenac sodium in improving hematological parameters (CRP, ESR). In addition, celecoxib was more effective at reducing complications than diclofenac sodium in the treatment of KOA (OR = 0.34, 95% CI = 0.20 to 0.59, *P* ≤ 0.001).

Systematic reviews and meta-analyses are secondary studies based on published literature, which inevitably have some limitations. First, most of the included studies were found in the Chinese literature, except for three in other countries (Britain, Sweden, and India), so the results of the studies lack a broader representation. Second, there are differences in the measurement methods of a small number of results included in the studies, which makes it difficult to interpret the results. In addition, the quality of the systematic reviews was affected by the different trial durations, which indicates that similar research designs should be more standardized in the future. There are differences in the gender ratio of the included studies, which impacts the results of the systematic review. The quality of life scale should be added as an important outcome measure to evaluate the efficacy of celecoxib and diclofenac sodium in future studies. In addition, a complete report of iatrogenic adverse events or complications should be conducted in clinical studies.

The safety of drugs is an important problem in clinical applications. KOA is prone to occurring in middle-aged and elderly people. Most NSAIDs can stimulate the gastrointestinal tract, induce ulcers, and affect renal function and platelets. Because selective COX-2 inhibitors do not affect the activity of COX-1, they can reduce the gastrointestinal events caused by the inhibition of COX-1. One of the concerns about COX-2 selective inhibitors is that they increase cardiovascular risk. Traditional NSAIDs (such as aspirin) have a certain protective effect on the cardiovascular system. They selectively inhibit platelet COX-1 and, thus, reduce TXA synthesis, but they do not affect the production of anti-aggregation PG and prostacyclin (COX-2-mediated) by endothelial cells. COX-2 selective inhibitors reduce the synthesis of prostacyclin in endothelial cells, thus increasing the rate of thrombosis, which leads to cardiovascular events. There are four types of renal damage caused by NSAIDs: acute renal insufficiency, acute interstitial nephritis, chronic interstitial nephritis, and renal papillary necrosis. Currently, the mechanism of renal damage from NSAIDs has not been fully elucidated but may be related to the following factors: 1) inhibition of prostaglandin synthesis and reduction of local vasodilator factors in the kidney, which may lead to renal vasoconstriction and decrease in renal blood flow; 2) inhibition of renal tubular cell enzyme activity due to direct tubular toxicity; 3) medullary arteriosclerosis, leading to renal papillary necrosis; and 4) drug-induced allergic reactions.

## Conclusions

Based on the current evidence, celecoxib has a positive impact on improving the treatment effect of KOA by reducing pain, improving hematological indicators (CRP, ESR), and reducing the incidence of complications. However, the limitations of study methodology have a certain impact on the reliability of the conclusions. To accurately evaluate the effectiveness and safety of celecoxib, more high-quality RCTs with unified measurements and guidance are needed in the future.

## Authors’ Contributions

Conceived and designed the experiments: Hetao Huang and Jun Liu; performed the experiments: Minghui Luo, Haodong Liang, Jianke Pan, and Weiyi Yang; analyzed the data: Lingfeng Zeng, Guihong Liang, Senrong Hou, and Jinlong Zhao; wrote the paper: Hetao Huang and Minghui Luo.

## Notes

Funding sources: This study was supported by grants from the National Natural Science Foundation of China (No.81974574, No. 81873314), Key Scientific Research Platforms and Research Projects of Universities in Guangdong Province (No. 2018KQNCX041), China Postdoctoral Science Foundation (No. 2018M633036), Project of State Administration of Traditional Chinese Medicine of China (No. 20201129), Medical Science Research Foundation of Guangdong Province (No. A2020105, No. B2019091), Project of Guangdong Provincial Department of Finance (No. [2018]8, No. [2014]157), and the Science and Technology Research Project of Guangdong Provincial Hospital of Chinese Medicine (No. YN2019ML08, YN2015MS15).
